# The complete chloroplast genome sequence and phylogenetic position of *Lilaeopsis chinensis* (L.) Kuntze (Apiaceae)

**DOI:** 10.1080/23802359.2021.1984332

**Published:** 2021-10-06

**Authors:** Xiaoai Fang, Peng Li, Ling Zhao

**Affiliations:** Department of Pharmacy, Xi'an International University, Xi’an, China

**Keywords:** *Lilaeopsis chinensis* (L.) Kuntze, chloroplast genome, Illumina sequencing, phylogenetic analysis, Apiaceae

## Abstract

*Lilaeopsis chinensis* (L.) Kuntze is a traditional Chinese medicinal plant for treating diuresis, abdominal pain and eczema. In this study, its complete chloroplast genome was assembled from the whole genome Illumina sequencing data. The circular genome is 153,162 bp long, and comprises a pair of inverted repeat regions (IRs, 25,072 bp each), a large single-copy region (LSC, 84,288 bp) and a small single-copy region (SSC, 18,730 bp). It encodes a total of 113 genes (79 protein-coding, 30 tRNA and 4 rRNA genes), with 19 of them occurring in double copies. Introns were detected in 11 protein coding genes (PCG) and 6 tRNA genes. The nucleotide composition is asymmetric (30.9% A, 19.2% C, 18.4% G and 31.5% T) with an overall A + T content of 62.4%. Phylogenetic analysis challenged the traditional taxonomic framework of the family Apiaceae, and indicated that *Lilaeopsis chinensis* (L.) Kuntze is closely related to *Hydrocotyle verticillata*.

Chloroplasts are generally believed to originate from cyanobacteria, which are produced through endosymbiosis in plant. This organelle contains chloroplast-specific components and regulates starch storage, sugar synthesis, the biosynthesis of key cellular materials (fatty acids, pigments, vitamins and amino acids). To formulate efficient prevention, management and control strategies of invasive species, it is essential to gain insight into their taxonomy, biogeography and genetics, and chloroplast DNA has proven powerful for such purposes (Fama et al. 2002; Oduor et al. 2015; Mukherjee et al. 2016).

*Lilaeopsis chinensis* (L.) Kuntze (*Hydrocotyle chinensis* L., 1753) is a deciduous plant belonging to the family Apiaceae. It is widespread throughout Hunan, Sichuan and Yunnan in China. It is a perennially terrestrial herb with short-creeping rhizomes, pale brown stripes and rachises, and narrowly obovate ultimate segments. Except as a beautiful ornamental plant, it is also a traditional Chinese medicinal plant for treating diuresis, abdominal pain, eczema, etc. (Xu et al. 2013; Xiong et al. 2019). In this study, base on the chloroplast (cp) genome of *Lilaeopsis chinensis* (L.) Kuntze, we attempt to provide additional valuable data for the phylogenetic study of Apiaceae in the future.

Fresh leaves was obtained from Chengdu (104.07E, 30.67 N; Sichuan, China), and the herbarium specimen deposited at Pharmaceutical Laboratory in Xi’an International University (https://www.xaiu.edu.cn/, Ling Zhao, hdyxyz1619@163.com) under the voucher number LC932677. Total genomic DNA was isolated from approximately 100 mg of fresh leaves of *Lilaeopsis chinensis* (L.) Kuntze using the DNeasy Plant MiniKit (Qiagen, CA, USA). After the detection of DNA purity and integrity, high-quality DNA was used to library construction and sequenced using Illumina Noveseq with paired-end 150 strategy. Genomic DNA was used for sequencing by the Illumina HiSeq X Ten Sequencing System (Illumina, CA, USA). The raw sequencing data were quality-trimmed with Geneious R11 (Biomatter Ltd., Auckland, New Zealand), and were then used for the assembly of chloroplast genome with MITObim v1.9 (Hahn et al. 2013). The chloroplast genome of *Hydrocotyle verticillata* Thunb. (HM596070) was used as initial reference. After the assembly, the trimmed raw data were mapped to the assembled chloroplast sequence to check the assembly quality and coverage.

The complete cp genome of *Lilaeopsis chinensis* (L.) Kuntze has a total sequence length of 153,162 bp, and exhibits a typical quadripartite structure of the large (LSC, 84,288 bp) and small (SSC, 18,730 bp) single-copy regions, separated by a pair of inverted repeat regions (IRs, 25,072 bp) . It encodes a total of 113 genes (79 protein-coding, 30 tRNA and 4 rRNA genes). 17 genes are partially or completely duplicated, including 11 PCG genes (*atpF*, *clpP*, *ndhA*, *ndh*B, *pet*B, *rpl*2, *rpl*16, *rpo*C1, *rps*12, *rps16* and *ycf*2) and 6 tRNA genes (*trn*A-UGC, *trn*G-UCC, *trn*I*-*GAU*, trn*K*-*UUU*, trn*L*-*UAA and *trn*V-UAC). The nucleotide composition is asymmetric (30.9% A, 19.2% C, 18.4% G and 31.5% T) with an overall A + T content of 62.4%.

To identify the *Lilaeopsis chinensis* (L.) Kuntze phylogenetic position of within the family Apiaceae, the Bayesian Inference (BI) phylogenetic tree was reconstructed with the MRBayes V3.1.1 (Ronquist and Huelsenbeck 2003) and Topali V2.5 (Milne et al. 2009) based on the complete cp genome of *Lilaeopsis chinensis* (L.) Kuntze and other 36 species (Figure 1), we can find that *Hydrocotyle sibthorpioides*, *Hydrocotyle verticillata* and *Lilaeopsis chinensis* (L.) Kuntze formed a monophyletic clade. Besides, *Lilaeopsis chinensis* (L.) Kuntze (MW387999) is closely related to *Hydrocotyle verticillata* (HM596070). Our findings will contribute to phylogenomic study of the Apiaceae in the future, and provide important information for the development and utilization of characteristic plant resources.

**Figure 1.**
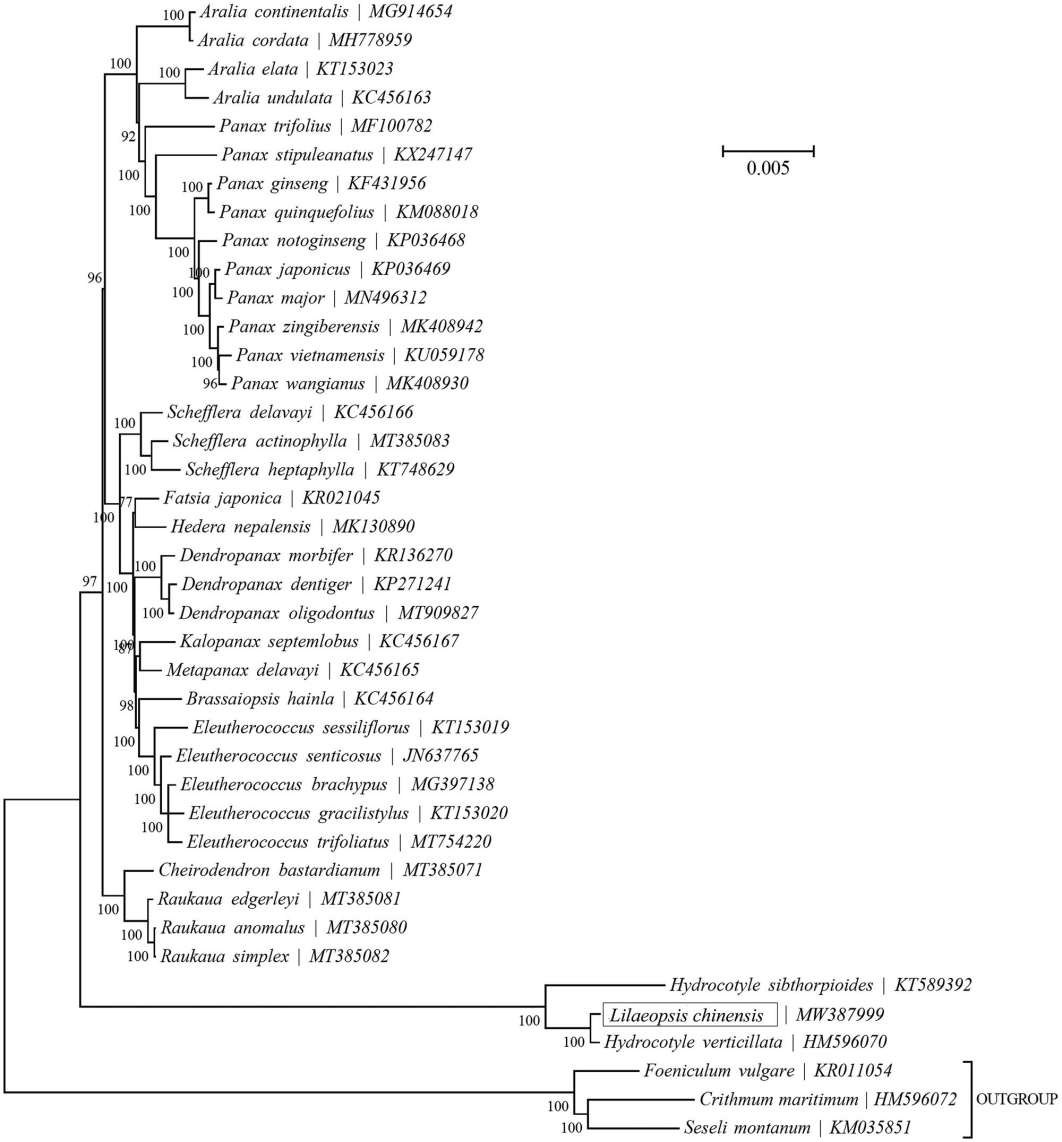
Bayesian Inference (BI) phylogenetic analysis inferred from 37 complete chloroplast genomes. The bootstrap values were based on 1000 resamplings and are placed next to the branches.

## Data Availability

The data that support the findings of this study are openly available in the US National Center for Biotechnology Information (NCBI database) at https://www.ncbi.nlm.nih.gov/, reference number: MW387999. The associated BioProject, SRA, and Bio-Sample numbers are PRJNA728361, SRR14494993 and SAMN19077562, respectively.
